# Neurological manifestations in thrombotic microangiopathy: Imaging features, risk factors and clinical course

**DOI:** 10.1371/journal.pone.0272290

**Published:** 2022-09-21

**Authors:** José Thiago de Souza de Castro, Simone Appenzeller, Marina Pereira Colella, Gabriela Yamaguti-Hayakawa, Erich Vinícius De Paula, Joyce Annichinno-Bizzachi, Fernando Cendes, Reis Fabiano, Fernanda Andrade Orsi

**Affiliations:** 1 School of Medical Sciences, University of Campinas, (UNICAMP), Campinas, São Paulo, Brazil; 2 Department of Radiology, University of Campinas, (UNICAMP), Campinas, São Paulo, Brazil; 3 Rheumatology Unit, School of Medical Science, University of Campinas, Campinas, São Paulo, Brazil; 4 Thrombosis and Hemostasis Unit, Hematology and Hemotherapy Center, University of Campinas, (UNICAMP), Campinas, São Paulo, Brazil; 5 Discipline of Hematology and Hemotherapy, Department of Internal Medicine, School of Medical Sciences, University of Campinas, (UNICAMP), Campinas, São Paulo, Brazil; 6 Department of Neurology, School of Medical Sciences, University of Campinas, Campinas, São Paulo, Brazil; 7 Department of Clinical Pathology, School of Medical Sciences, University of Campinas, (UNICAMP), Campinas, São Paulo, Brazil; Ohio State University, UNITED STATES

## Abstract

**Background and purpose:**

Thrombotic microangiopathy (TMA) is a group of microvascular occlusive disorders that presents with neurological involvement in up to 87% of the cases. Although the central nervous system (CNS) is an important target organ in TMA, the role of neurological manifestations in the disease clinical course is not well established. In this study, we described the neurological manifestations and CNS radiological aspects in patients with a first, acute TMA event. We also examined the association between severe neurological involvement and adverse clinical outcomes in TMA.

**Methods:**

A cohort of patients diagnosed with a first TMA event between 1995 and 2016 was included, their medical charts and imaging tests were retrospectively evaluated.

**Results:**

A total of 49 patients were included, 85.7% were women and the mean age was 36.5 years-old (SD 13.0). Neurological manifestations were described in 85.7% of the patients, most of them (88%) were considered severe and consisted of confusion, compromised sensorimotor function, stupor, seizures, and personality change. Imaging tests were performed in 62% of the patients with neurological manifestations and detected acute CNS lesions, such as posterior reversible encephalopathy syndrome, hemorrhagic and ischemic stroke were observed, in 7 (27%) of them. While the need for intensive care unit admission was greater and longer among patients with severe neurological manifestations, the number of plasma exchange sessions, the total duration of hospitalization and in-hospital death were similar between groups.

**Conclusions:**

Severe neurological manifestations are common in first TMA events and are responsible for a worse disease presentation at admission. While the effect of neurological manifestations on acute TMA clinical course seems to be modest, these manifestations may have an important impact on the development of chronic cognitive impairment, which highlights the need for proper diagnosis and treatment.

## Introduction

Thrombotic microangiopathy (TMA) is a group of microvascular occlusive disorders characterized by thrombocytopenia and hemolysis associated with different degrees of intravascular thrombi formation, which can lead to ischemia in the brain, kidney, and other organs [1, 2]. Although rare, TMAs are predominantly life threatening conditions that require urgent diagnosis and management [3].

TMA is a group of disorders characterized by thrombocytopenia (platelet count below 150 x 109/L), microangiopathic hemolytic anemia (presence of schistocytes in the blood smear, elevated reticulocyte count, low haptoglobin levels, and negative direct antiglobulin test), and organ dysfunction (elevated lactate dehydrogenase and signs of end organ damage due to ischemia) [3–7]. There are different types of TMA, such as thrombotic thrombocytopenic purpura (TTP), hemolytic-uremic syndrome (HUS), thrombotic microangiopathies associated with pregnancy, autoimmune diseases, malignant hypertension, infections, drugs, cancer/chemotherapy and transplantation [3–7]. The differences between these TMA are primarily their pathological mechanisms, but there is also variation with regards to the target organs affected by the disease. As an example, the central nervous system (CNS) and heart are predominantly affected in TTP, while the kidney is mainly affected in HUS.

Thrombotic thrombocytopenic purpura (TTP) and hemolytic-uremic syndrome (HUS) are the most common types of TMA [8]. TTP and HUS events are mostly idiopathic, but in some cases the conditions are associated with underlying diseases, such as infections, neoplasia, autoimmune disorders [8–10]. Although rare, there are also cases of congenital TTP [11]. Although the involvement of the central nervous system (CNS) is more frequent in TTP [1, 12], in which it is detected in 63% to 87% of the cases [13–17], up to 20% of HUS patients also present with some degree of neurological manifestations [18].

Several pathophysiological mechanisms have been implicated with TMA neurological involvement, such as endothelial injury, thrombus formation, hemorrhage, and posterior reversible encephalopathy syndrome (PRES) [16]. Neurological manifestations vary from mild to severe, which includes headache, focal neurological symptoms (according to the anatomical site of damage), delirium, seizures, and coma [13–15, 18].

Although the CNS is a prevalent target organ in TMA [16], the role of neurological manifestations in the clinical course of TMA is not well established. Therefore, this study aimed to describe the neurological manifestations and radiological aspects of the CNS in patients with a first, acute TMA event. We also evaluated whether severe neurological involvement is associated with adverse clinical outcomes in first TMA events.

## Methods

### Participants in the study and TMA treatment

In this observational study, we evaluated a cohort of TMA adult patients treated at the UNICAMP Clinical Hospital (University of Campinas—Brazil) between 1995 and 2016. Data was retrospectively retrieved from medical charts. The patients were identified in an internal TMA database. TMA diagnosis was performed by the hematology team and attending physicians. The diagnosis was based on the association of thrombocytopenia and hemolytic anemia with negative antiglobulin test and schistocytes in the peripheral blood smear. Secondary causes of TMA were investigated with the following parameters: hepatitis and HIV serology, anti-nuclear test, chest, and abdominal imaging evaluation with CT. Past medical history regarding medications in use and comorbidities was also obtained. Most patients had clinical suspicions of TTP, and were treated as TTP; however we could not confirm this diagnosis, or exclude other TMA, because the ADAMTS13 (13th member of a disintegrin-like and metalloprotease with thrombospondin type 1 motifs) test was not available. Despite being necessary to confirm TTP diagnosis, the ADAMTS13 test was not available for most patients, because the test is not reimbursed by the Brazilian Health System.

After diagnosis, patients were treated with daily therapeutic plasma exchange (TPE), consisting of one plasma volume exchange using fresh frozen plasma, and corticosteroid (prednisone 1 mg/kg per day). In cases where platelet count did not reach values above 100.000 mm^3^ within 7 days, volume TPE was doubled and/or other immunosuppressant drugs were prescribed. During the study period, the two changes in therapy practices occurred: i. TPE was stopped when platelet reached 100.000/uL until 2007 and after 2008 TPE was stopped only when platelet reached levels above 150.000/uL; ii. Second line immunosuppressive treatment consisted of vincristine and splenectomy until 2009 and after 2010 rituximab was incorporated as a second line therapy. Patients requiring renal replacement therapy at the diagnosis were transferred to the Nephrology unit. Patients with neurological manifestations were evaluated by a neurologist, and brain CT or magnetic resonance imaging (MRI) was performed.

### Clinical and neurological evaluation

Data about demographic characteristics (sex, age and ethnic group), number of prior TMA episodes, underlying disease, time elapsed from the onset of symptoms until treatment, pregnancy, platelet count, indirect bilirubin levels, reticulocyte count, coagulation parameters, creatinine levels, neurological manifestations, radiological data, number of TPE sessions, duration of hospitalization, relapse and death was retrospectively retrieved from the medical charts. For this study, we considered the laboratory data obtained upon admission.

We used the clinical and laboratory data to calculate the patients’ PLASMIC score. The PLASMIC score is a tool applied for the prediction of ADAMTS13 deficiency that aids in timely treatment, especially when ADAMTS13 is not promptly available. The PLASMIC score uses clinical and laboratory data that are scored, following a pre-definition, on a scale that varies between 0 and 7 [14, 15, 33]. The clinical and laboratory parameters of the score are: platelet count, variables related to hemolysis, the diagnosis of cancer or prior transplant, values of mean corpuscular volume, international normalized ratio (INR) and serum creatinine. Scores from 0 to 4 indicate low risk, score 5 indicates intermediate risk and scores 6 or 7 indicate high risk of ADAMTS13 deficiency [19].

We considered as neurological manifestations the following signs or symptoms: headache, confusion, personality changes, sensorimotor loss, seizures, stupor or coma, ischemic stroke, and hemorrhagic stroke. The presence of headache alone was considered a mild neurological involvement, while the other manifestations were considered severe neurological involvement. This classification was in accordance with that used by Lotta et al. [20]. Data on neurological manifestations were retrieved from the medical charts and imaging tests.

The study was approved by the Institutional Ethics Committee of the School of Medical Sciences of the University of Campinas (CAAE 02465018.3.0000.5404). The need for written informed consent was waived based on the study design.

### Statistical analysis

Baseline characteristics were denoted as number and frequencies (percentage) when categorical variables and as mean and standard deviation (SD) when they were continuous variables. Median and interquartile range (IQR) were used to express continuous variables that did not achieve normal distribution. Fisher exact test was used to compare categorical data. To compare continuous data at baseline, the independent t-test was used when variables were normally distributed, and the Mann-Whitney test when variables were not normally distributed. Data were analyzed using The SAS System for Windows (Statistical Analysis System), version 9.4. SAS Institute Inc, 2002–2012, Cary, NC, USA. P values less than 0.05 were considered statistically significant.

## Results

A total of 49 patients presenting with a first TMA event were included. Tables 1 and 2 demonstrate the baseline demographic features, clinical and neurological manifestations during hospitalization. Most patients were women (85.7%), and the mean age was 36.5 years-old (SD 13.0). Two patients had HIV infection, two had systemic lupus erythematosus, one had cancer (osteosarcoma) and eight were pregnant or postpartum. There were no cases of drug or transplant-associated TMA. ADAMTS13 activity was tested in only 13 patients (26.5%), and the results (all below 10%) confirmed the diagnosis of TTP. By retrospectively applying the PLASMIC score, as previously described [14, 15], we observed that all patients scored 5 or above. The PLASMIC score was 5 in 9%, 6 in 56.8% and 7 in 34% of the patients, suggesting that most of them may have had a TTP episode. Neurological (85.7%) and abdominal (59.2%) symptoms were the most common clinical manifestations of TMA patients, followed by bleedings (55%) and renal impairment (26.5%). Five patients (10.2%) died during hospitalization.

**Table 1:** Baseline characteristics of 49 patients with a first TMA event by their neurological status.

Parameters	TMA patients (n = 49)	Mild or no neurological manifestations (n = 13)	Severe neurological manifestations (n = 36)	P
Women, n (%)	42 (85.7)	12 (92.3)	30 (83.3)	0.68
Age at diagnosis, mean (SD)	36.5 (13.0)	33.2 (16.2)	37.8 (11.6)	0.27
Underlying disease, n (%)	6 (12.2)	2 (15.4)	4 (11.1)	0.33
Types underlying diseases:				
HIV	2	0	2	
Cancer	1	0	1	
SLE	3	2	1	
History of transplant	0 (0)	0 (0)	0 (0)	
Pregnancy or postpartum, y (%)	8 (16.3)	4 (30)	4 (11.1)	0.184
Neurological manifestation, n (%)	42 (85.7)	6	36	
Type of neurological manifestation, n (%)				
Headache only, n (%)	6 (12.2)		6 (12.2)	
Confusion, n (%)	26 (53.1)		26 (53.1)	
Personality Changes, n (%)	8 (16.3)		8 (16.3)	
Compromised Sensorimotor Function, n (%)	23 (46.9)		23 (46.9)	
Seizures, n (%)	12 (24.5)		12 (24.5)	
Stupor, n (%)	21 (42.9)		21 (42.9)	
Abnormal MRI or CT scan of the brain (%) *	7 (26.9)	1 (16.7)	10 (32)	0.001
CNS involvement in imaging tests				
Hemorrhagic stroke	2		2	
Ischemic stroke	4		4	
PRES	1		1	

*CNS imaging was available for 26 patients.

Abbreviations: TMA: thrombotic microangiopathy. CT: computed tomography; Y: Yes; N: No; CNS: central nervous system; HIV: human immunodeficiency virus; SLE: systemic lupus erythematosus; NA: not available; MRI: magnetic resonance imaging; ECG: electrocardiogram; SD: standard deviation. PRES: posterior reversible encephalopathy syndrome.

**Table 2:** Clinical course during hospitalization of 49 patients with a first TMA event by their neurological status.

Parameters	TMA patients (n = 49)	Mild or no neurological manifestations (n = 13)	Severe neurological manifestations (n = 36)	P
Cardiac involvement *, n (%)	8 (16.3)	2 (15.4)	6 (16.7)	1.0
Abdominal symptoms (pain, nausea, vomiting), n (%)	29 (59.2)	5 (38.5)	24 (66.7)	0.10
Renal impairment**, n (%)	13 (26.5)	3 (23.1)	10 (27.8)	0.92
Minor bleedings ***, n (%)	27 (55.1)	8 (61.5)	19 (52.8)	1.0
Major bleedings ****, n (%)	5 (10.2)	0 (0)	5 (13.9)	0.35
Thrombosis, n (%)	1 (2.0)	0 (0)	1 (2.8)	1.0
Infection, n (%)	30 (61.2)	8 (61.7)	22 (61.1)	1.0
Number of TPE sessions, mean (SD)	13.6 (9.9)	13.4 (10.9)	14.0 (8.95)	0.83
Days in intensive care unit, mean (SD)	3.75 (5.2)	0.4 (1.4)	7.1 (9.0)	0.008
Duration of hospitalization in days, mean (SD)	27.5 (11.7)	26.2 (10.3)	28.9 (13.1)	0.56
Death during hospitalization, n (%)	5 (10.2)	1 (7.7)	4 (11.1)	1.00

*chest pain, arrhythmias, pericarditis, ECG changes;

**creatinine > 2 mg/dL during hospitalization

***petechiae, ecchymoses, oozing from venipuncture sites;

****CNS or retinal bleeding. Abbreviations: SD: standard deviation; TPE: plasma exchange therapy.

**Fig 1:**
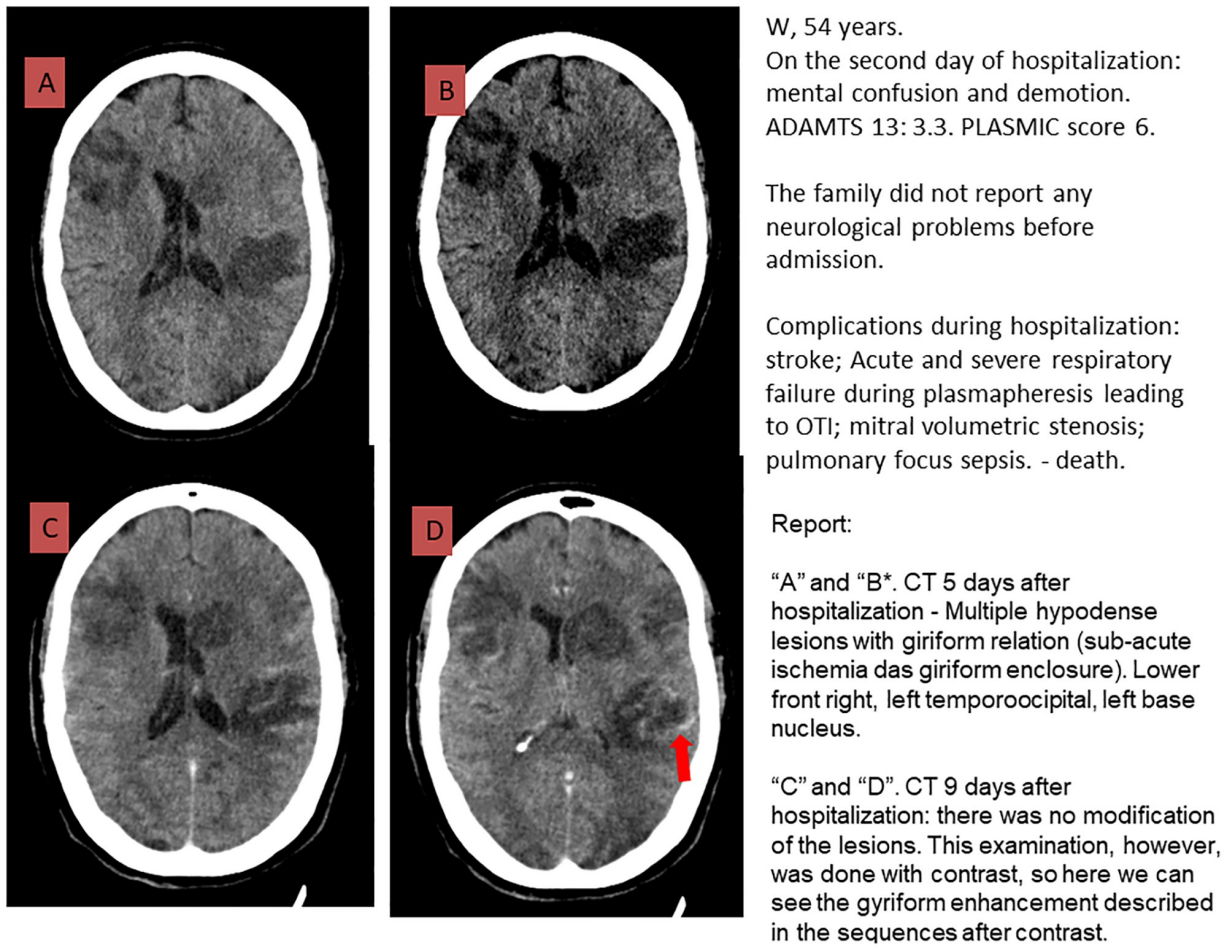


**Fig 2:**
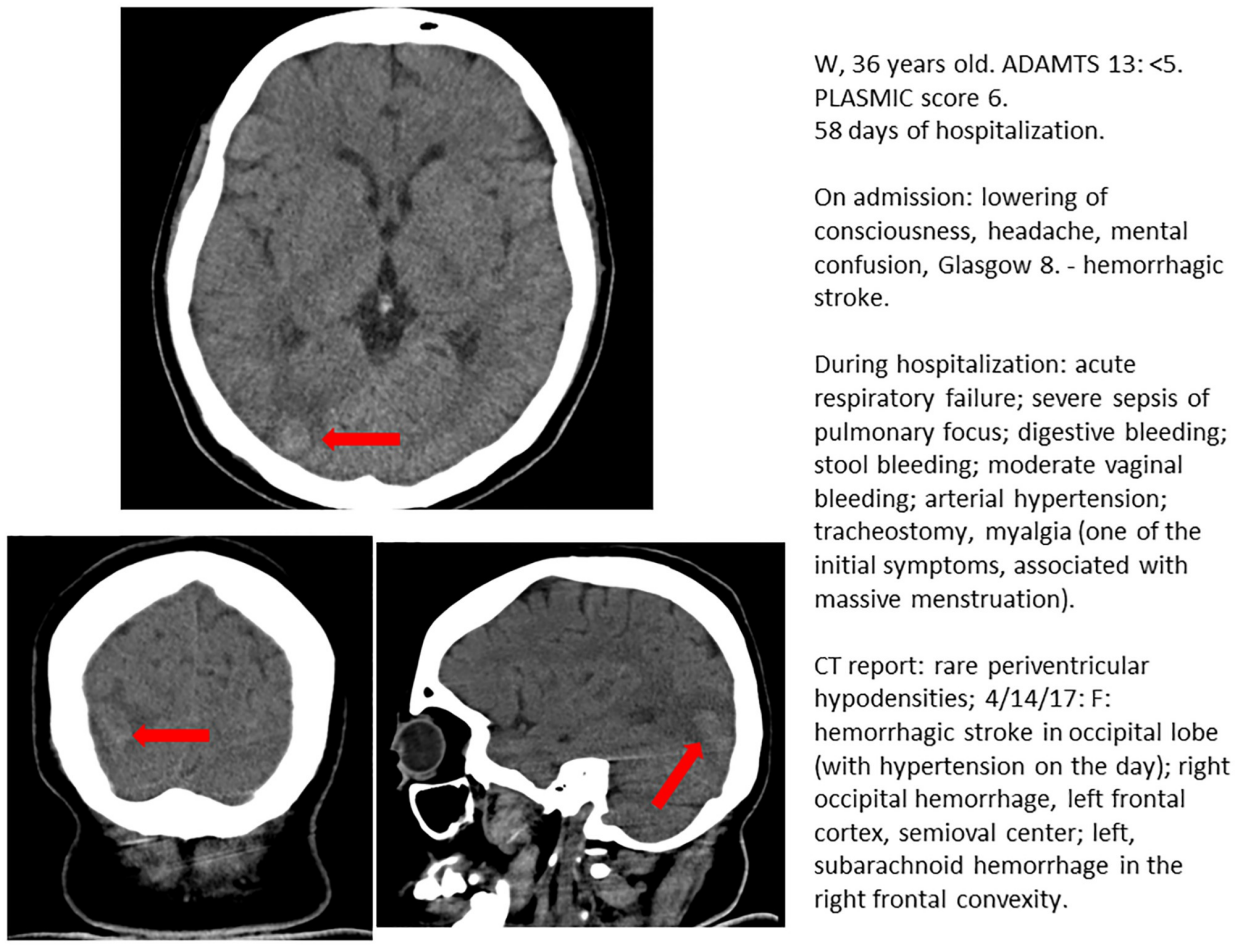


Neurological manifestations were described in 42 (85.7%) TMA cases and most of them were considered severe (confusion, personality changes, sensorimotor loss, seizures, stupor or coma, ischemic stroke, or hemorrhagic stroke). CT and/or MRI were performed on 26 patients during hospitalization (62% of the patients with neurological manifestations). Details of CT or MRI findings are provided in Table 3. Twenty-three patients were submitted to CT only, one patient was submitted to MRI and 2 patients performed both CT and MRI. Acute neurological findings were observed in the CT images of 7 patients; ischemic stroke was observed in 4 patients, hemorrhagic stroke in 2 patients, and posterior reversible encephalopathy syndrome in 1 patient. Figs 1 and 2 illustrate the cerebral lesions. All patients who had acute lesions depicted on CT had one or more focal neurological manifestations (headache, paresthesia, mental confusion, or seizure).

**Table 3:** CNS lesions detected on CT or MRI during first TMA events.

Sex	Age	Description in neuroimaging exams
F	18	CT after 3 days of hospitalization: hemorrhagic stroke (bilateral occipital lesions, and left temporoparietal lesion).
F	23	CT on the fourth day of hospitalization: normal; CT after 15 days of hospitalization: Hypodense lesions compatible with ischemic stroke.
F	29	CT performed 7 days after hospitalization showing subarachnoid hemorrhage (Fisher 4).
F	31	CT performed 25 days after hospitalization, during exacerbation, showing hypodensity and retraction of the occipital lobe parenchyma.
F	36	CT at the date of hospitalization rare periventricular hypodensities; MRI after 15 days of hospitalization: occipital lobe hemorrhagic stroke (with hypertension on the day); right occipital hemorrhage, consistent with PRES. Associated with hypertension and acute renal failure.
F	54	CT 3 days after hospitalization: Multiple hypodense lesions with gyriform enhancement (subacute ischemia), in the inferior frontal gyrus, right and left temporooccipital region and left basal ganglia.
F	68	CT after 6 days of hospitalization: bifrontal cortical ischemic lesions, in the right precentral gyrus, in the middle frontal gyrus, and in the right parietal lobe.

CT: computed tomography, MRI: magnetic resonance. PRES: posterior reversible encephalopathy syndrome. CT: computed tomography. MRI: magnetic resonance imaging. CNS: central nervous system.

Next, we compared patients with and without severe neurological manifestations at the diagnosis. We considered the following as severe neurological manifestations: the presence of confusion, personality changes, sensorimotor loss, seizures, stupor, or coma, ischemic or hemorrhagic stroke. The presence of isolated headache was considered a mild neurological manifestation. Table 1 demonstrates the baseline demographic and clinical features of the TMA patients according to their neurological status. Sex, age, the diagnosis of underlying diseases and the time elapsed from the beginning of the symptoms until TPE was started did not differ between patients with and without severe neurological manifestations. In terms of laboratory parameters, patients with severe neurological manifestations had a lower platelet count at diagnosis as compared to patients without these manifestations. There was also a trend for higher INR and lower creatinine levels among patients with severe neurological manifestations as compared to those without. These results are shown in Table 4.

**Table 4:** Laboratory parameters upon hospital admission.

Parameters	Mild or no neurological manifestations	Severe neurological manifestations	P
	(n = 13)	(n = 36)	
Time elapsed from first symptoms to TPE (days), mean (SD)	19.0 (16.9)	17.0 (11.7)	0.77
Platelet count (x10^9^/mL), mean (SD)	22.9 (21.1)	14.7 (6.3)	0.04
Reticulocyte count (%), mean (SD)	8.7 (6.4)	9.7 (5.2)	0.56
Indirect bilirubin (mg/dL), mean (SD)	1.85 (1.29)	2.19 (1.58)	0.56
INR, mean (SD)	1.11 (0.08)	1.19 (0.13)	0.06
Creatinine (mg/dL), mean (SD)	1.84 (2.5)	1.04 (0.43)	0.07

TMA: thrombotic microangiopathy. SD: standard deviation. INR: (international normalized ratio); TPE: plasma exchange therapy.

Table 2 demonstrates the clinical outcomes of TMA patients with and without severe neurological manifestations. The need for intensive care unit (ICU) admission and the duration of ICU stay were higher in patients with severe neurological manifestations than in those with mild or no neurological manifestations (7.1 [SD 9.0] days vs. 0.4 [SD 1.4] days, P = 0.008). Despite the longer ICU stay among patients with severe neurological manifestations, the number of TPE sessions, the total duration of hospitalization and in-hospital deaths were similar between groups.

## Discussion

In this study, neurological manifestations occurred in most TMA patients at diagnosis and, in 73% of them, the neurological involvement consisted of severe manifestations, such as confusion, sensorimotor compromise, and stupor. These findings corroborate previous reports, as neurological manifestations during acute TMA events are common and have been reported by several authors [9, 10]. The most commonly reported neurological manifestations in previous studies are headache, seizure, dizziness, vertigo, visual changes, altered mental status, and altered conscious state [20–23]. These complications can evolve to posterior reversible encephalopathy syndrome (PRES), as reported by at least two studies [22, 24, 25], and sometimes to coma [26]. Most CNS lesions are characterized as hemorrhagic or ischemic cerebral infarctions [20, 26, 27, 29] and, therefore, TMA may be misdiagnosed as a case of stroke [28, 29]. Given that TMA is a medical emergency associated with neurological sequelae and a fatality rate of 20%, a quick and precise diagnosis is essential to prevent adverse outcomes [10].

Despite the well characterized neurological symptoms, the detection of CNS lesions by imaging exams is not always possible. Previous clinical studies reported that the proportion of abnormal CT exams varies from 0 to 25% [22] in TMA patients with neurological manifestations, while the proportion of abnormal MRI is 82% in the same population [3]. MRI was demonstrated to be more accurate and to detect lesions not identified by CT scan, such PRES and small strokes [22]. In our study, we observed that imaging tests (CT or MRI) were performed in 26 out of 42 (62%) patients with neurological manifestations and CNS lesions were observed in only 7 (26.9%) of these imaging tests. The reported lesions were ischemic stroke (4 cases), hemorrhagic stroke (2 cases) and PRES (1 case), which is in line with previous studies that reported that ischemia, hemorrhage, and PRES are the main CNS lesions in TMA. The abnormalities observed in CT scans or MRI justified the neurological manifestations presented by the patients. In addition, it reinforces the importance of performing neuroimaging evaluation during episodes of TMA, especially in the presence of neurological manifestation, since the pattern of alteration detected (hemorrhage or ischemia) guides to the appropriate therapy [22]. Although CT scan and MRI were not capable of depicting structural CNS damage in most of our patients with neurological manifestations, MRI is the gold standard method to demonstrate structural abnormalities that may cause lasting cerebral dysfunction. A prospective study in those patients with demonstrable structural changes, compared to those without, may better reflect the impact of the neurologic outcome in these patients.

In addition, we also evaluated the clinical course of TMA in patients with severe neurological manifestations, as compared to those with mild or no neurological manifestations. We observed that patients with severe neurological manifestations presented with a more severe form of TMA at diagnosis, with lower platelet count and greater need for ICU admission than those with mild or no neurological manifestations. Despite the worse clinical presentation at diagnosis, the clinical course during hospitalization was similar to that observed in patients with mild or no neurological manifestations, since the number of TPE sessions needed for TMA remission, the duration of hospitalization and death did not substantially differ between the groups. Similar findings have been recently reported by other studies, in which neurological manifestations were not associated with disease exacerbation or mortality [29–31]. Neurological manifestations during an acute TMA event, however, seem to have an important impact on the development of cognitive disorders in the future, leading to depression, anxiety, and intellectual impairment [32].

There are limitations of our study that must be discussed. First, the diagnosis of TTP could not be confirmed in most cases because the ADAMTS13 activity test was performed in only 26.5% of the patients. The lack of ADAMTS13 tests is explained by the fact that, despite being necessary to confirm TTP diagnosis, the test is not easily available in the real world, particularly in low- and middle-income countries. For that reason, the PLASMIC score has been used to predict low ADAMTS13 activity [19–32]. The use of PLASMIC score in this study allowed us to suspect that most of our patients had TTP even though ADAMTS13 results were not available. This score, however, has some limitations. As an example, platelet count may be overestimated in splenectomized patients because they are less likely to present with platelet counts below <30 × 109/L than non-splenectomized patients. Prior platelets or fresh frozen plasma transfusion may also affect the platelet count, interfering with the final score [20, 21, 33]. Despite these limitations, the PLASMIC score is a valuable tool in case testing for ADAMTS13 is not possible. Among our patients, 44 (89.8%) had a score of 6 or 7 and the remaining had a score of 5, suggesting that most of them had TTP. Second, neuroradiological evaluation was not available for all patients, and most patients performed only CT. As discussed above, MRI is more accurate to characterize acute lesions than CT, as they usually demonstrate restricted diffusion on diffusion-weighted images and may depict other brain lesions. MRI is also recommended to detect structural abnormalities in PRES. Therefore, if MRI had been performed for all patients with neurological manifestations, the characteristics of CNS lesions detected in this study could have been different. Finally, the sample size of this study is low, however it reflects the rarity of TMA. Despite the limitations, our results could picture the neurological manifestations in TMA, describing in detail their clinical and radiological aspects.

## Conclusion

In this study, we confirmed that severe neurological manifestations are prevalent in first TMA events and are responsible for a worse disease presentation at admission. Despite the high prevalence of the neurological symptoms, imaging tests, particularly CT scans, are not able to completely detect the CNS lesions, possibly because some patients present with a cerebral dysfunction, not structural damage, and MRI should be performed when possible. While neurological manifestations were not associated with poorer response to treatment and death, their role in the development of chronic neurological disorders, such as depression and dementia, warrants the need for proper diagnosis and treatment.

## Supporting information

S1 Data (XLSX)
